# An MFS-Domain Protein Pb115 Plays a Critical Role in Gamete Fertilization of the Malaria Parasite *Plasmodium berghei*

**DOI:** 10.3389/fmicb.2019.02193

**Published:** 2019-09-20

**Authors:** Fei Liu, Qingyang Liu, Chunyun Yu, Yan Zhao, Yudi Wu, Hui Min, Yue Qiu, Ying Jin, Jun Miao, Liwang Cui, Yaming Cao

**Affiliations:** ^1^Department of Immunology, College of Basic Medical Sciences, China Medical University, Shenyang, China; ^2^Department of Internal Medicine, Morsani College of Medicine, University of South Florida, Tampa, Tampa, FL, United States; ^3^The First Hospital of China Medical University, Shenyang, China; ^4^Liaoning Research Institute of Family Planning, Shenyang, China

**Keywords:** *Plasmodium berghei*, yeast protein expression, gamete, gamete interaction, transmission-blocking

## Abstract

Sexual reproduction is an essential process in the *Plasmodium* life cycle and a vulnerable step for blocking transmission from the human host to mosquitoes. In this study, we characterized the functions of a conserved cell membrane protein P115 in the rodent malaria parasite *Plasmodium berghei* ANKA. Pb115 was expressed in both asexual stages (schizonts) and sexual stages (gametocytes, gametes, and ookinetes), and was localized on the plasma membrane of gametes and ookinetes. In *P. berghei*, genetic deletion of *Pb115* (Δ*pb115*) did not affect asexual multiplication, nor did it affect gametocyte development or exflagellation of the male gametocytes. However, mosquitoes fed on Δ*pb115*-infected mice showed 74% reduction in the prevalence of infection and 96.5% reduction in oocyst density compared to those fed on wild-type *P. berghei*-infected mice. The Δ*pb115* parasites showed significant defects in the interactions between the male and female gametes, and as a result, very few zygotes were formed in ookinete cultures. Cross fertilization with the male-defective Δ*pbs48/45* line and the female-defective Δ*pfs47* line further indicated that the fertilization defects of the Δ*pb115* lines were present in both male and female gametes. We evaluated the transmission-blocking potential of Pb115 by immunization of mice with a recombinant Pb115 fragment. *In vivo* mosquito feeding assay showed Pb115 immunization conferred modest, but significant transmission reducing activity with 44% reduction in infection prevalence and 39% reduction in oocyst density. Our results described functional characterization of a conserved membrane protein as a fertility factor in *Plasmodium* and demonstrated transmission-blocking potential of this antigen.

## Introduction

Malarial incidence has significantly decreased in recent years due to a range of actions, including the deployment of insecticide-treated nets, indoor residual spraying and artemisinin-based combination therapies (Bhatt et al., 2015). Nevertheless, recent World Health Organization reports showed that the global progress toward malaria elimination has stalled (World Health Organization [WHO], 2018). To achieve global elimination of malaria, an integrated malaria control strategy and novel interventions are needed, which may include measures that interrupt and inhibit disease transmission.

During the complex life cycle of malaria parasite, sexual stages are obligative for the transmission of the parasite from the human host to the mosquitoes. Male and female gametocytes formed in the human blood, after ingestion by a female anopheline mosquito, undergo gametogenesis to form gametes, which then fuse to form zygotes. Later, zygotes mature into ookinetes, which penetrate the midgut epithelium to differentiate into oocysts.

Transmission from the human host to the mosquito represents a major population bottleneck in the life cycle of the parasite (Vaughan, 2007). Transmission-blocking vaccines (TBVs) target this bottleneck by eliciting antibodies that inhibit the fusion of the male and female gametes (pre-fertilization) and maturation from zygotes to ookinetes (post-fertilization). To date, a substantial number of antigens expressed during sexual development have been studied for their TBV potentials (Nikolaeva et al., 2015; Wu et al., 2015; Delves et al., 2018), but only three antigens, namely the pre-fertilization antigens Pfs48/45 (Kocken et al., 1993; Outchkourov et al., 2008; Chowdhury et al., 2009) and Pfs230 (Williamson et al., 1993; Lee et al., 2017; Tachibana et al., 2019), as well as the post-fertilization antigen Pfs25 (Kaslow et al., 1988; Shimp et al., 2013; Talaat et al., 2016; Sagara et al., 2018) and its ortholog Pvs25 in *Plasmodium vivax* (Tsuboi et al., 1998; Sagara et al., 2018), have been extensively investigated as lead vaccine candidates. However, all these TBV candidates are conformational antigens containing multiple disulfide bridges, and the production of correctly folded antigens is an important challenge (Sauerwein and Bousema, 2015). Therefore, continuous efforts on TBV antigen discovery are warranted.

The deciphering of *Plasmodium* genomes has provided an unprecedented opportunity for large-scale functional studies toward a better understanding of the fundamental developmental biology of the parasite (Gardner et al., 2002; Hall et al., 2005). This has also fueled the functional screening of potential TBV antigens during ookinete development in the more genetically amenable rodent parasite *Plasmodium berghei* (Ecker et al., 2008). Using a similar strategy, we identified Pb115, an evolutionarily conserved, putative membrane protein that is expressed in both asexual and sexual stages of the malaria parasites. Through genetic manipulation studies in *P. berghei*, we found that the mutant parasites lacking *Pb115* had a major defect in ookinete formation, resulting in transmission failure to the mosquitoes. Genetic crosses revealed that both male and female gametes require this protein for gamete recognition and attachment. Immunization of mice with the recombinant Pb115 protein induced strong antibody responses that effectively blocked formation of ookinetes, highlighting the TBV potential of this protein.

## Materials and Methods

### Mice, Parasites, and Mosquitoes

Six-to-eight-week old female BALB/c mice were purchased from Beijing Animal Institute (Beijing, China). The *P. berghei* ANKA strain 2.34 was maintained by serial passage and used for challenge infections as described previously (Blagborough and Sinden, 2009). Adult female *Anopheles stephensi* mosquitoes (Hor strain) were reared in an insectary under 25°C, 50–80% humidity and a 12 h light/dark cycle, and fed 10% (w/v) glucose solution-soaked cotton balls. All animal experiments were approved by the animal ethics committee of China Medical University.

### Sequence Analysis

To identify genes encoding potential ookinete surface proteins, we searched the malaria database PlasmoDB^1^ for proteins expressed in ookinetes with a putative signal peptide or at least one transmembrane domain. We identified a gene *PBANKA_0931000*, which encodes a putative protein of 115 kDa, henceforth designated as Pb115. The sequences of its orthologs in other *Plasmodium* species were retrieved from PlasmoDB and aligned using the ClustalW multiple sequence alignment program.

### Generation of Transgenic Parasites

The HA-tagging and gene-knockout (KO) targeting vectors for *Pb115* (PbGEM-290856 and PbGEM-290848) were acquired from PlasmoGEM (Wellcome Trust Sanger Institute, Cambridge, United Kingdom). HA-tagged (pb115-HA) and *Pb115* KO (Δ*pb115*) parasite lines were generated by homologous recombination as previously described (Braks et al., 2006). To obtain schizonts for transfection, parasitized red blood cells (RBCs) were collected from mice at day 4 after infection and cultured at 37°C overnight in the culture medium [RPMI 1640, 20% (v/v) fetal calf serum, and 50 mg/L penicillin and streptomycin] at 0.5 mL blood/50 mL medium. The culture was then fractionated on a 55% (v/v) Nycodenz cushion to purify the schizonts (Dempsey et al., 2013). Linearized plasmids were transfected into the schizonts using Nucleofector II (Lonza, Stockholm, Sweden), and the parasite suspension was intravenously injected into two BALB/c mice through the tail vein. At 24 h after injection, mice were treated with pyrimethamine (0.07 mg/ml) via drinking water for 3–4 days. PCR was used to identify the desired integration events in transfected parasites using integration-specific primers (Supplementary Table S1). For tagging, the HA tag was inserted at the C-terminus of the *pb115* gene, and for KO, the open reading frame of *pb115* was replaced with an *hdhfr* expression cassette. Parasites were cloned by limiting dilution. Primers for 5′ or 3′ recombination fragments and integration-specific PCR are listed in Supplementary Table S1.

### Expression of Recombinant Pb115 (rPb115) and Immunization

A 205 amino acid (aa) fragment of the Pb115 (aa 756–960) was expressed in the yeast *Pichia pastoris*. Briefly, the Pb115 DNA fragment was amplified using primers CGTACGTACAAAATATTAAAACTATTTTTTCATCATTT and TTGCGGCCGCTCTTGCCTTCCAATATATGGTAAATATTAG (restriction sites underlined) and cloned into the pPIC3.5K(+His) plasmid. A positive yeast strain was cultured in 1 L of BMMG medium and rPb115 expression was induced with methanol. Yeast cells were collected by centrifugation and lysed using an ATS high pressure homogenizer (ATS Engineering Inc., Germany). Recombinant proteins were purified with the Ni-NTA column (Novagen, Germany). The quality of purified protein was analyzed by SDS-PAGE.

To generate antisera against Pb115, 6–8-week old female BALB/c mice (*n* = 5) were immunized with 50 μg of purified rPb115 emulsified with complete Freund’s adjuvant. Mice were then given two booster injections at 2-week intervals with 25 μg of protein, each emulsified with incomplete Freund’s adjuvant. Mice in the control group (*n* = 5) were immunized with adjuvant formulations in phosphate buffered saline (PBS, pH 7.0). Two weeks after the final immunization, blood was collected from mice via cardiac puncture and allowed to clot at room temperature to obtain the antisera. An enzyme-linked immunoassay (ELISA) was used to analyze the antibody titers as previously described (Chan et al., 2014).

### Western Blot

Western blots were performed with parasite lysates and protein fractions of different developmental stages. Purified schizonts, gametocytes, and ookinetes were treated with 0.15% saponin (Sigma) in PBS for 10 min on ice. Parasites were collected by centrifugation and washed once with PBS. Parasite proteins were collected following repeated extraction in PBS containing 1% Triton X-100, 2% SDS and protease inhibitors (Roche, Basel, Switzerland) for 30 min at room temperature. Lysates of non-infected erythrocytes and Δ*pb115* ookinetes were used as negative controls. For subcellular protein fractionation, plasma membrane (PM) and cytoplasm of the parasites were separated using the Minute^TM^ Plasma Membrane Protein Isolation and Cell Fractionation Kit (Invent Biotechnologies Inc., United States) according to the manufacturer’s instructions. Protein concentration was determined using the BCA method, and equal amounts of protein lysates or cellular fractions (10 μg) were separated in 6% SDS-PAGE gels. For Western blots, proteins were transferred to a 0.22 μm polyvinylidene difluoride membrane (Bio-Rad, Hercules, CA, United States). The membrane was blocked with 5% non-fat milk in TBS buffer containing 0.1% Tween 20 (TBST) for 2 h, and then probed with anti-rPb115 antisera at 1:1000 or the anti-HA monoclonal antibody (mAb) at 1:1000 (Invitrogen, Carlsbad, CA, United States) for 2 h. Mouse anti-PbHsp70 antiserum (1:1000) was used as a control to estimate protein loading. After washing three times with TBST, bound primary antibodies were detected with HRP-conjugated goat anti-mouse antibodies (Invitrogen) diluted 1:10,000 in TBST. After three washes with TBST, proteins on the blot were visualized using a Pierce ECL Western Blotting Kit (Thermo Fisher Scientific). Band intensities were measured by ImageJ and the relative expression levels of Pb115 protein were normalized against those of PbHsp70 for the corresponding stages.

### Indirect Immunofluorescence Assay

For Indirect Immunofluorescence Assay (IFA), HA-tagged or wild-type (WT) parasites were fixed with 4% paraformaldehyde and 0.0075% glutaraldehyde in PBS for 20 min at room temperature and rinsed with 50 mM glycine in PBS. To differentiate internal and external protein localizations, cells were either permeabilized with 0.1% Triton X-100/PBS for 10 min or directly processed without permeabilization. To liberate schizonts and gametocytes from the enveloping RBC and parasitophorous vacuole membranes, schizonts and gametocytes were treated with 0.05% saponin/PBS for 3 min at 37°C prior to fixation. Without further membrane permeabilization, cells were washed in PBS and then treated with 0.1 mg/ml of sodium borohydride in PBS for 10 min to reduce the free aldehyde groups (Tonkin et al., 2004). After blocking with PBS containing 3% BSA/PBS at 37°C for 1 h, WT and Pb115-HA parasites were incubated with mouse anti-rPb115 antisera (1:500) and mouse anti-HA mAb (1:500, Invitrogen), respectively, at 37°C for 1 h. Cells were co-incubated with rabbit antisera against PbMSP1, Pbg377, α-tubulin and PSOP25 as stage-specific markers for schizonts, female gametocytes/gametes, male gametocytes/gametes, and ookinetes, respectively. Then the slides were washed three times with PBS and incubated with polyclonal Alexa Flour 488-conjugated goat-anti-mouse IgG secondary antibodies (1:500, Invitrogen) and Alexa Flour 555-conjugated goat-anti-rabbit IgG secondary antibodies (1:500, Cell signaling), respectively, at 37°C for 30 min. Parasite nuclei were stained with 4, 6-diamidino-2-phenylindole (DAPI, Invitrogen) at a final concentration of 1 μg/mL. As negative controls, WT ookinete smears were incubated mouse anti-HA mAb (1:1000, Invitrogen) and control sera obtained from mice immunized with adjuvant emulsified in PBS as primary antibodies, or secondary antibodies alone. Slides were mounted with ProLong^®^ Gold antifade reagent (Invitrogen) and observed under a Nikon C2 fluorescence confocal laser scanning microscope.

### Phenotypic Analysis of Δ*pb115*

To study the functions of Pb115 during the *Plasmodium* development, mice in each group were injected with either 1 × 10^6^ WT *P. berghei-* or Δ*pb115*-infected RBCs (iRBCs) (Clone 1 and Clone 2). Parasitemia and mortality of mice were monitored daily. To determine the effect on parasite sexual development, mice were pre-treated with 0.2 mL of 6 mg/mL phenylhydrazine for 3 days before injection of iRBCs. On day 3 after infection, gametocytemia and the gametocyte sex ratio were determined by Giemsa-stained tail blood smears (Guttery et al., 2014). At least 100 mature gametocytes were quantified as males and females to determine the gametocyte sex ratio (Liu et al., 2018). Exflagellation centers of male gametocytes and male–female gametes interactions were quantified as previously described (Tewari et al., 2010). In short, 10 μL tail blood from infected mice was added to 40 μL standard ookinete medium (RPMI 1640 with 50 mg/L penicillin, 50 mg/L streptomycin, 100 mg/L neomycin, 20% [v/v] heat-inactivated fetal calf serum [FCS], pH 8.0). Fifteen minutes after induction of gamete formation at 25°C, 1 μL of culture was placed on a coverslip (Matsunami Glass Ind., Ltd., Japan) and analyzed under a light microscope (40× objective). The exflagellation centers were counted as an exflagellating male gametocyte with four red blood cells in 10 min. Male–female gametes interactions were counted as the males attached females for more than 3 s during a period of 20 min in 10 fields (40× objective) (van Dijk et al., 2010). To count the macrogamete numbers, ookinete culture was set up as described above. After incubation at 25°C for 15 min, 1 μL of culture were placed on a coverslip and female gametes were stained with mouse anti-Pbs21 antibody (1:1000) without permeabilization. The numbers of female gametes in 1 μL of culture were counted under a fluorescence microscope (100× oil objective). The culture was further incubated at 19°C; 1 μL of culture was taken out at 2 h and 24 h to determine the number of zygotes and ookinetes, respectively (Tewari et al., 2010). To determine whether defects of Δ*pb115* lines were female- or male-specific, *Pbs47* KO (Δ*pbs47*) and *Pbs48/45* KO (Δ*pbs48/45*) lines were used in an *in vitro* cross-fertilization assay as described (van Dijk et al., 2010; Zhu et al., 2019). At 3 days post infection, equal numbers of mature gametocytes of different clones were mixed and incubated for 24 h. The numbers of ookinetes were counted as described above (100× oil objective).

To determine the subsequent development, mosquito feeding experiment was performed with mice that were infected three days earlier with either the WT or Δ*pb115 P. berghei*. Four-day-old female *A. stephensi* mosquitoes (∼100/mouse) starved for 6 h were allowed to feed on infected mice for 30 min. Unfed mosquitoes were then removed and fed mosquitoes were maintained at 19–22°C and in 50–80% relative humidity. Ten days after feeding, up to 50 mosquitoes were dissected in each group. The midguts of mosquitoes were removed and stained with 0.5% mercurochrome (Sigma-Aldrich). Oocysts were counted to determine the prevalence (number of infected mosquitoes) and intensity of infection (number of oocysts per positive midgut).

### Quantification of Transmission-Blocking Activity

Transmission-blocking activity (TBA) of anti-rPb115 antisera was estimated using both *in vitro* ookinete conversion and *in vivo* mosquito feeding assays (Kou et al., 2016). In short, phenylhydrazine pre-treated mice were injected with 1 × 10^6^ WT iRBCs. Three days post-infection, parasitemia was counted by Giemsa staining, and 10 μL gametocyte-infected blood were mixed with 90 μL ookinete culture medium containing 10% anti-Pb115 mouse sera or control sera (immunized with adjuvants only) incubated at 19°C for 2 h to count zygote numbers and 24 h to count ookinete numbers. Mosquito-feeding experiments were carried out as described previously (Zheng et al., 2017). Immunized mice with rPb115 protein or control mice were pre-treated with 0.2 mL of 6 mg/mL phenylhydrazine for 3 days. Five mice from each group were injected with 1 × 10^6^ WT iRBCs, and mosquitoes were fed on immunized mice at day 3 after infection. Ten days after blood meal, the prevalence and intensity of infection in mosquitoes were determined.

### Statistical Analysis

Statistical comparison between groups (IgG levels, parasitemia, gametocytemia, and ookinete numbers) was performed by Student’s *t* test using the GraphPad Prism software. The intensity of mosquito infection (oocysts/midgut) was analyzed using the Mann–Whitney *U* test, while infection prevalence was analyzed by Fisher’s exact test using SPSS version 21.0. Survival of mice infected with WT or Δ*pb115* parasites was compared by using the Kaplan–Meyer’s method. All data were from three independent experiments.

## Results

### P115 Is Highly Conserved Among *Plasmodium* Species

*PBANKA_0931000* (Pb115) is among a group of genes with the following features: conserved in *Plasmodium* genomes; sexual-stage expression; and containing the sequence for a putative signal peptide or at least one transmembrane domain (Zheng et al., 2016). The *Pb115* gene is located on chromosome 9 and encodes a protein of 978 aa with a predicted size of 115 kDa. As shown in the predicted protein features in PlasmoDB, the predicted protein contains a membrane lipoprotein lipid attachment site at the N-terminus and a major facilitator superfamily (MFS) general substrate transporter domain (*P* value 1.05e-14) near the C terminus (Supplementary Figure S1). The encoded protein contains 12 transmembrane domains, six within the MFS domain. Multiple sequence alignment showed that this protein is highly conserved among *Plasmodium* species (Supplementary Figure S2).

### Pb115 Is Expressed in Both Asexual and Sexual Stages

**FIGURE 1:**
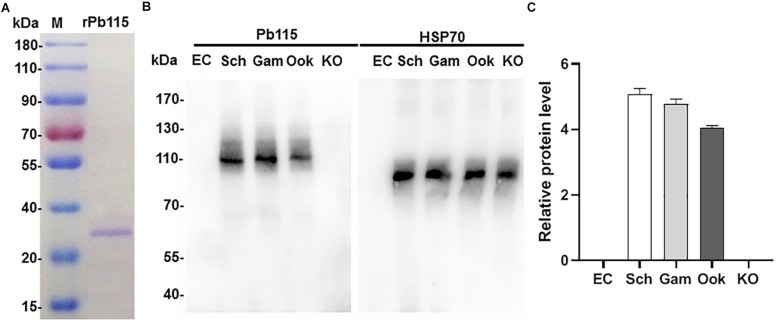
Recombinant Pb115 protein purification and detection of Pb115 expression during parasite development. **(A)** Purified recombinant Pb115 protein was subjected to electrophoresis on a 12% polyacrylamide gel, and the proteins were stained with Coomassie Brilliant Blue. M: PageRuler Prestained Protein Ladder in kDa. **(B)** Representative Western blot of Pb115 in asexual- and sexual-stage parasites. Lysates of schizonts (Sch), gametocytes (Gam), ookinetes (Ook), and Δ*pb115* parasites (KO) at 10 μg/lane were probed with mouse anti-Pb115 sera (1:500). The Δ*pb115* parasite protein extract was a mixture of 7 μg asexual-stage proteins and 3 μg ookinete proteins. Protein loading was estimated by Western blot with the anti-Hsp70 sera (1:1000). Non-infected erythrocytes (EC) were used as negative control. **(C)** The Pb115 relative protein levels in schizonts (Sch), gametocytes (Gam), ookinetes (Ook), and KO parasites were estimated by using ImageJ analysis of replicate Western blots shown in **(B)**. Data were normalized against the PbHsp70 bands of the respective stages.

Expression of the ortholog of *Pb115* in *Plasmodium falciparum* (*PF3D7_1117000*), as demonstrated by RNA-seq analysis, was mainly in schizonts during the asexual intraerythrocytic development cycle (Plasmodb.org). The *PF3D7_1117000* mRNA was also detected during sexual development and was almost equally abundant in male and female gametocytes (Lasonder et al., 2016). To study Pb115 protein expression during *P. berghei* development, we generated a recombinant Pb115 fragment, which was used to generate antibodies directed against the protein. For this, the 205 aa MFS domain from aa 756 to aa 960 (Supplementary Figure S1) was expressed in yeast and purified using the Ni-NTA column. SDS-PAGE of the purified recombinant protein (rPb115) showed a relatively homogenous protein band with a molecular weight of ∼23 kDa (Figure 1A), which agreed with the predicted size of the MFS domain. The rPb115 protein was used to immunize 6–8-week-old female BALB/c mice. Two weeks after the third immunization, the immune sera contained a significantly higher antibody titer against the rPb115 fragment than the adjuvant control sera (Supplementary Figure S3). The antisera were used to probe lysates from purified *P. berghei* schizonts, gametocytes and cultured ookinetes in Western blots. Compared with the erythrocyte lysate control, the anti-rPb115 antisera specifically recognized a protein of approximately 115 kDa in the three developmental stages, consistent with the predicted molecular size of Pb115. Using HSP70 for protein loading control, Pb115 showed similar expression levels in all these three stages examined (Figure 1B). ImageJ analysis of the band intensities in three replicated Western blots showed a slight lower Pb115 abundance in ookinetes than in schizonts and gametocytes (Figure 1C).

To further verify that the anti-rPb115 sera indeed identified the Pb115 protein, we generated a *P. berghei* parasite line with the endogenous Pb115 tagged with HA at its C-terminus. Correct fusion of the *Pb115* gene with the HA tag was confirmed by diagnostic integration PCR (Supplementary Figure S4). Western blotting with the anti-HA mAb identified a protein band of ∼115 kDa, similar to the size of the protein identified by the anti-Pb115 antisera. No specific protein bands were detected in lysates from non-infected erythrocytes and WT ookinetes (Supplementary Figure S4). Similarly, Pb115-HA protein level in ookinetes was slightly lower than those in schizonts and gametocytes.

### Pb115 Is Localized on the Plasma Membrane of Gametes and Ookinetes

The presence of multiple transmembrane domains in Pb115 suggests that this protein might be associated with membrane structures in the parasites. To detect membrane association of Pb115, we separated the parasite PM with the cytoplasm and performed Western blots using the anti-Pb115 antisera. In schizonts and gametocytes, Pb115 was detected in both the cytoplasm and PM fractions, with Pb115 appearing more abundant in the PM fractions (Supplementary Figure S5). Interestingly, in ookinetes, Pb115 was mainly detected in the PM fraction. Using the same procedure, we evaluated the subcellular distribution of Pb115 in the Pb115-HA parasite line using the anti-HA mAb. The results obtained using both the WT and Pb115-HA lines were highly comparable (Supplementary Figure S5).

**FIGURE 2:**
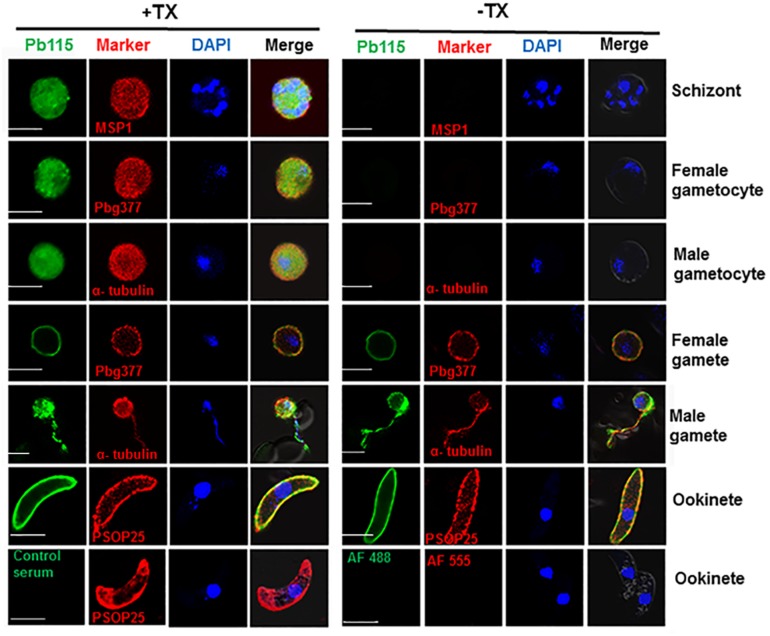
Representative IFA images of Pb115 in WT *P. berghei* with (+TX) or without (–TX) membrane permeabilization. Parasites were incubated with anti-Pb115 sera (1: 500) as the primary antibodies (green). The parasites were also labeled with antibodies against the marker proteins for different stages (PbMSP1 for schizonts, Pbg377 for female gametocytes and gametes, α-tubulin for male gametocytes and gametes, and PSOP25 for ookinetes). Nuclei were stained with DAPI (1 μg/mL) (blue). The bottom panel shows two negative controls: WT ookinetes (+TX) labeled with the control serum or only with the secondary antibodies (AF488, Alexa Fluor 488, and AF555, Alexa Fluor 555). Merge, Alexa Flour 488 + Alexa Flour 555 + DAPI. Bar, 5 μm. Data are representatives of three independent experiments.

We further examined Pb115 expression and localization in more detail by IFA. In WT parasites, IFA with the anti-rPb115 sera detected Pb115 protein expression in schizonts, gametocytes, gametes, and ookinetes (Figure 2), whereas fluorescence was almost undetectable in rings, trophozoites and sporozoites (data not shown). As negative controls, IFA with control mouse sera or without primary antibodies did not produce fluorescence signals (Figure 2). In both schizonts and gametocytes, Pb115 fluorescence dispersed throughout the cytoplasm, sometimes with a speckled appearance. In female gametes, the Pb115 signal was found to be associated with the PM only, whereas in male gametes, it was associated with both the flagella and the residual body (Figure 2). Consistent with the Western analysis showing primary association of Pb115 with the PM in ookinetes, IFA also showed association of Pb115 with the PM of ookinetes with the signals partially being co-localized with those of PSOP25. To differentiate internal from external membrane localization, IFA was performed without membrane permeabilization. In this case, schizonts and gametocytes were not labeled, indicating that the Pb115 protein was not localized outside of the membrane of the iRBC (Figure 2). During gamete development, the female gametes showed a similar localization pattern regardless of membrane permeabilization status, demonstrating external membrane localization of the Pb115 on female gametes. Similarly, in male gametes, the fluorescent signals were detected on both residual bodies and flagella-like male gametes. Again, Pb115 was clearly localized on the PM of ookinetes (Figure 2). IFA with developmental stages of the Pb115-HA line using anti-HA mAb showed highly similar patterns of Pb115 localization (Supplementary Figure S6).

To further demonstrate the PM association of Pb115 in schizonts and gametocytes, parasites were liberated from the enveloping RBC and parasitophorous vacuole membranes via mild saponin treatment. Subsequent IFA showed that Pb115 was localized at the peripheral PM in both male and female gametocytes (Supplementary Figure S7A). This localization pattern was further confirmed using the Pb115-HA line probed with the anti-HA mAb (Supplementary Figure S7B). Collectively, these data indicated that Pb115 was expressed from schizonts to ookinetes, and Pb115 retained PM localization from gametes through ookinete development. Given that the Pb115 antisera were generated against the C-terminus and HA was also tagged to the C-terminus, these localization patterns suggest that the Pb115 C-terminus was external to the PM.

### Pb115 Is Needed for Gamete Attachment

**FIGURE 3:**
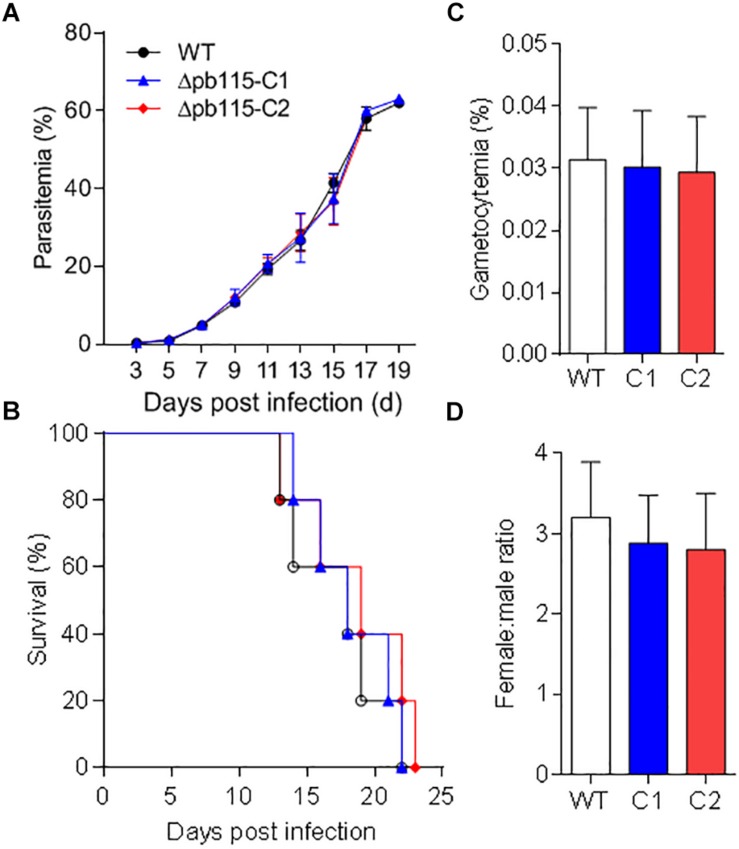
Functional analysis of Pb115 during parasite blood stages. Mice were infected with *P. berghei* or Δ*pb115* parasites Clone 1 (C1) and Clone 2 (C2). **(A)** Growth curve of asexual stages (10 mice per group). **(B)** Survival rates of mice infected with different parasite strains (10 mice per group). **(C)** Gametocytemia on day 3 after infection by different parasite strains (five mice per group). **(D)** Female: male gametocyte ratios on day 3 after infection by WT *P. berghei* and Δ*pb115* clone 1 and 2 (five mice per group). The data are from three biological experiments and are presented as the mean ± SD in each group.

**FIGURE 4:**
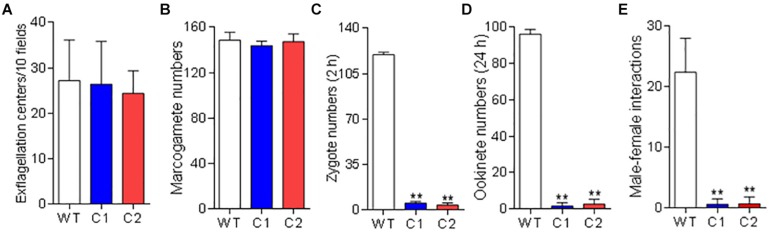
Function analysis of Pb115 in sexual stages. *P. berghei* or Δ*pb115* parasites (Clone 1 and Clone 2)-infected mouse blood on day 3 after infection was incubated in the ookinete culture medium. **(A)** Exflagellation centers of male gametocytes in 10 microscopic fields under a 40× objective. **(B)** Numbers of female gametes in 1 μL of ookinete culture at 15 min post activation **(C)** Numbers of zygotes formed at 2 h during *in vitro* ookinete culture. **(D)** Numbers of ookinetes formed at 24 h during *in vitro* ookinete culture. Data are presented as the mean ± SD for five mice in each group. **(E)** Male–female gamete interactions. Numbers of male gametes attached to females for more than 3 s during 20 min of observation. Data are presented as the mean ± SD from three experiments. ^∗∗^ indicates *P* < 0.01 for comparison with WT parasite (*t* test).

To investigate the function of Pb115 in *P. berghei* development, the *pb115* gene was knocked out by homologous recombination. Integration-specific PCR was used to confirm the KO lines (Supplementary Figures S8A,B). In addition, the lack of Pb115 protein expression in a KO line was confirmed by Western blot analysis (Figures 1B,C) and IFA (Supplementary Figure S8C). Two Δ*pb115* lines were selected from two independent transfection experiments and used for phenotype analysis. The effect of the *pb115* KO on parasite development was examined during blood-stage infection and in mosquitoes. Although Pb115 was expressed in schizonts, deletion of *pb115* did not affect asexual blood-stage multiplication of the parasites (*P* > 0.05; Figure 3A), nor did it impact the survival of infected mice (*P* > 0.05, Kaplan–Meier’s survival analysis; Figure 3B). With regard to sexual stages, there were no differences in gametocytemia and sex ratio between the WT and Δ*pb115* lines (*P* > 0.05; Figures 3C,D). Furthermore, the Δ*pb115* parasites showed no defects in gametogenesis, as the numbers of exflagellation centers and macrogamete numbers were all comparable between the WT and the Δ*pb115* lines (*P* > 0.05; Figures 4A,B). Using *in vitro* assays, we then evaluated whether *pb115* KO affected the fertilization and subsequent sexual development process. During our *in vitro* culture of ookinetes, the zygote numbers formed at 2 h and ookinete numbers at 24 h in the two Δ*pb115* clones suffered similar levels of reduction (∼95%) compared to the WT parasites (*P* < 0.01; Figures 4C,D), suggesting that Δ*pb115* parasites were defective in fertilization. This finding was further reinforced by results from mosquito feeding experiments, where *A. stephensi* mosquitoes were allowed to feed on WT *P. berghei*- or Δ*pb115-*infected mice and midgut oocysts were quantified. Mosquitoes fed on WT *P. berghei-*infected mice had 88–96% prevalence of infection in three experiments, whereas mosquitoes fed on Δ*pb115-*infected mice had infection prevalence ranging from 12 to 16%, a reduction by 74% (*P* < 0.001; Table 1). Similarly, a drastic reduction in oocyst density was observed in mosquitoes fed on Δ*pb115-*infected mice (*P* < 0.001; Table 1 and Supplementary Figure S9A). Specifically, the mean number of oocysts per midgut was 118 in mosquitoes fed on WT-infected mice as compared to <4 on average in those fed on Δ*pb115*-infected mice.

**TABLE 1:** Phenotypic comparison between Δ*pb115* and WT *P. berghei* in mosquitoes.

**Experiment**	**Parasites**	**% infected mosquitoes (infected/dissected)**	**% reduction in prevalence^a^**	**Mean% reduction^b^**	**Oocyst density (mean ± SD)^c^**	**% reduction in oocyst density^d^**	**Mean% reduction^e^**
I	WT	88.0(44/50)			131.3 ± 33.2		
	Δ*pb115*	12.0(6/50)	76.0		2.3 ± 1.0	83.3	
II	WT	92.0(46/50)			102.9 ± 18.3		
	Δ*pb115*	16.0(8/50)	76.0		6.3 ± 1.7	93.9	
III	WT	96.0(48/50)			120.4 ± 22.0		
	Δ*pb115*	16.0(8/50)	80.0	74^∗∗∗^	3.3 ± 1.3	97.5	96.5^∗∗∗^

*WT- and* Δ*pb115-infected mice were fed to mosquitoes and prevalence of infection and oocyst density were compared between the two groups. ^*a*^% Reduction of prevalence = % prevalence_*wt*_ − % prevalence*_△_*_Pb115_; ^*b*^Fisher’s exact test; ^∗∗∗^*P* < 0.001; ^*c*^The number of oocysts per midgut (mean ± SD); ^*d*^% Reduction in oocyst density = (mean oocyst density_*wt*_ − mean oocyst density*_△_*_Pb115_)/mean oocyst density_*wt*_ × 100%; ^*e*^Mann–Whitney *U* test; ^∗∗∗^*P* < 0.001.*

**FIGURE 5:**
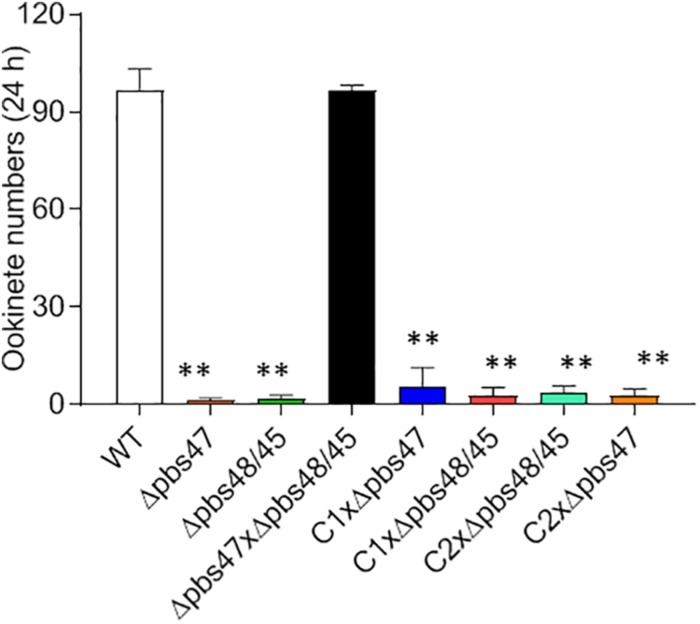
*In vitro* cross-fertilization studies. Δ*pb115* clone C1 and C2 and parasite lines that produce only fertile male (Δ*pbs47*) or only fertile female (Δ*pbs48/45*) gametes were incubated in different combinations during *in vitro* ookinete culture. The numbers of ookinetes formed were counted at 24 h. Data are from three independent experiments. ^∗∗^ indicates *P* < 0.01 for the comparison with WT *P. berghei* (*t* test).

To detect which step of the fertilization process was defective in the Δ*pb115* parasites, we performed detailed observations of the male–female gamete interactions under a phase contrast microscope. Male gametes from both WT and Δ*pb115* parasites exhibited similar motility and interaction with the RBCs, as evidenced by the similar numbers of exflagellation centers formed. In WT parasites, male–female attachments (for >3 s) were readily observed; on average 22 attachments were seen in five experiments (Figure 4E). By contrast, the number of gametes forming male–female attachments in the Δ*pb115* parasite lines was drastically reduced (five observed in eight experiments) (*P* < 0.01; Figure 4E), indicating that Δ*pb115* gametes were defective in recognition and attachment. To determine whether the resultant defects in gamete attachment in Δ*pb115* were due to either the male or female gamete, we performed *in vitro* cross-fertilization experiments between Δ*pb115* gametocytes and Δ*pbs47* or Δ*pbs48/45* gametocytes. As found in earlier studies (van Dijk et al., 2010), both Δ*pbs47* and Δ*pbs48/45* lines showed a fertilization rate that was decreased by more than 99% compared to WT (Figure 5). Since it has been reported that Δ*pbs47* produces normal male but defective female gametes, whereas the Δ*pbs48/45* line produces normal female but defective male gametes, we performed *in vitro* cross-fertilization to confirm that each line only had defects in one sex. As expected, similar numbers of ookinetes were formed in the Δ*pbs47* × Δ*pbs48/45* cross as compared to the WT (Figure 5). However, cross-fertilization of Δ*pb115* with either Δ*pbs47* or Δ*pbs48/45* failed to generate an appreciable number of ookinetes as compared with cross-fertilization between Δ*pbs47* and Δ*pbs48/45* (*P* < 0.01; Figure 5), suggesting that *pb115* deletion affected both male and female gametes.

### Antibodies Against Pb115 Display Apparent TRA

**FIGURE 6:**
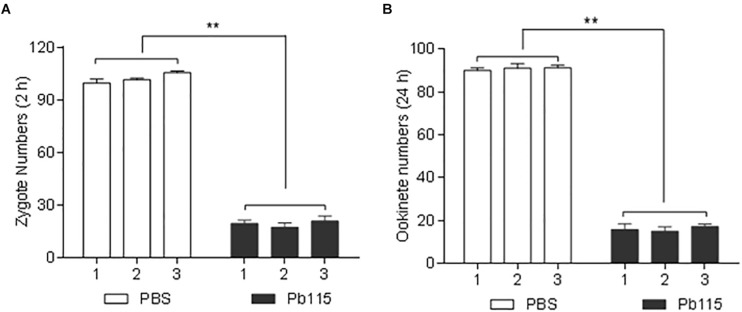
Quantification of TRA of the anti-Pb115 sera *in vitro*. Gametocyte-infected blood on day 3 after infection with *P. berghei* was incubated at 19°C in ookinete culture medium containing 10% anti-Pb115 mouse sera or control sera, and the numbers of zygotes and ookinetes formed at 2 and 24 h were counted. **(A)** Zygote numbers at 2 h. **(B)** Ookinete numbers at 24 h. The data are presented as the mean ± SD. ^∗∗^ indicates *P* < 0.01 for the comparison with the adjuvant control sera (*t* test).

Given that Pb115 was found localized on the PM of gametes and ookinetes, we wanted to test whether Pb115 had TRA. In the *in vitro* ookinete conversion assay, the number of zygotes formed during *in vitro* ookinete culture with the anti-rPb115 mouse sera was 5.3 times lower than those with the control sera (*P* < 0.01; Figure 6A). The number of ookinetes formed with the anti-rPb115 mouse sera at 24 h was 5.7 times lower than those with the control sera (*P* < 0.01; Figure 6B), suggesting that the immune sera had a major effect on reducing fertilization. In mosquito feeding assays, mice were immunized with rPb115 proteins and then infected with the WT *P. berghei*. Three days post infection, *A. stephensi* mosquitoes were allowed to directly feed on rPb115-immunized and control mice. Mosquitoes fed on control mice had an infection prevalence of 92–96% and oocyst density of 109.9–132.6 oocysts/midgut (Table 2). In comparison, mosquitoes fed on rPb115-immunized mice had infection prevalence of 44–52%, a reduction of 44% compared to mosquitoes fed on immunization control mice (*P* < 0.001, Fisher’s exact test, Table 2). Similarly, moderate levels of reduction (39%) in oocyst density (70–80.1 oocysts/midgut) were also observed in mosquitoes fed on rPb115-immunized mice as compared to those fed on control mice (*P* < 0.001, Mann–Whitney *U* test, Table 2 and Supplementary Figure S9B).

**TABLE 2:** *In vivo* evaluation of transmission blocking activity of Pb115.

**Experiment**	**Group**	**% infected mosquitoes (infected/dissected)**	**% reduction in prevalence^a^**	**Mean% reduction^b^**	**Oocyst density (mean ± SD)^c^**	**% reduction in oocyst density^d^**	**Mean% reduction^e^**
I	Control	92.0(46/50)			132.6 ± 21.0		
	rPb115	44.0(22/50)	48.0		70.0 ± 13.0	47.2	
II	Control	96.0(48/50)			109.9 ± 16.8		
	rPb115	52.0(26/50)	44.0		70.8 ± 11.7	35.1	
III	Control	92.0(46/50)			123.3 ± 21.2		
	rPb115	52.0(26/50)	40.0	44.0^∗∗∗^	80.1 ± 15.8	34.8	39.0^∗∗∗^

*Mosquitoes were fed on rPb115-immunized and control mice and the prevalence of infection and oocyst density were compared between the two groups. ^*a*^% Reduction of prevalence = % prevalence_*Control*_ − % prevalence_*rPb*115_; ^*b*^Fisher’s exact test; ^∗∗∗^*P* < 0.001; ^*c*^The number of oocysts per midgut (mean ± SD); ^*d*^% Reduction in oocyst density = mean oocyst density_*Control*_ − mean oocyst density_*rPb*115_)/mean oocyst density_*Control*_ × 100%; ^*e*^Mann–Whitney *U* test; ^∗∗∗^*P* < 0.001.*

## Discussion

Key fertilization factors discovered during functional studies of *Plasmodium* gamete membrane proteins include the male fertility factors Pbs48/45 and Pbs230 and female fertility factor Pbs47, which play critical roles in recognition and attachment to gametes of the opposite sex in the rodent parasite *P. berghei* (van Dijk et al., 2001, 2010). Interestingly, Pfs230 and Pfs47 seem to have divergent functions in *P. falciparum*: Pfs230 mediates erythrocyte binding (Eksi et al., 2006), whereas *Pfs47* is dispensable for female gamete fertility (van Schaijk et al., 2006) but is critical for immune evasion in mosquitoes (Molina-Cruz et al., 2013). Another male fertility factor, HAP2/GCS1, functions after the initial gamete recognition and attachment, since *PbHAP2* deletion lines have normal exflagellation and pairing of the male gametes with female gametes, but lack gamete fusion (Hirai et al., 2008; Liu et al., 2008).

In the current study, we identified a new gamete membrane protein, Pb115, whose deletion affected gamete fertility. The Pb115 protein has a conserved C-terminal MFS domain that is found in the MFS of transporter proteins, the largest superfamily of secondary carriers that are ubiquitously found in all branches of life with nearly 15,000 members. MFS transporters move a myriad of small molecules across biological membranes including sugars, peptides, deleterious substances, organic and inorganic ions (Yan, 2015). Based on the transport mode, MFS transporters are classified into uniporters (transporting a single substrate), symporters (transporting a substrate with a co-transporting ion or solute in the same direction), and antiporters (transporting a substrate with a co-transporting substrate in the opposite direction). They play diverse roles in homeostasis, metabolism and signal transduction (Law et al., 2008). It will be interesting to determine what, if any, compounds are transported by Pb115.

We learned from the current study that Pb115 is not essential for the intraerythrocytic stages – schizonts and gametocytes – despite its expression in these stages. In addition, gametogenesis in the absence of Pb115 appeared normal. Although the male gametes in the Δ*pb115* parasite showed similar motility to the WT parasites, and made contacts with the female gametes, most of the male–female interactions were transient (lasting less than 3 s) and futile. Furthermore, both male and female gametes were similarly affected by *pb115* deletion, as cross-fertilization with either Δ*pbs48/45* or Δ*pbs47* was not able to restore fertilization in the Δ*pb115* lines. It is noteworthy that despite the very low fertilization rates in the Δ*pb115* parasites, sporozoites dissected from salivary glands of Δ*pb115-*infected mosquitoes were equally infective to mice as the WT sporozoites (data not shown), suggesting the defects observed with Δ*pb115* were probably limited to gamete adhesion. Whether Δ*pb115* affects transport of essential molecules needed for male and/or female gamete functions or whether P115 impacts distribution of key fertility factors described above remains to be determined.

We have demonstrated using IFA that Pb115 was associated with the plasma membrane of gametes and ookinetes. Furthermore, Pb115 appeared to have a membrane localization conformation with its C-terminal domain residing outside the cell, given that the antibodies raised against MFS could detect Pb115 without membrane permeabilization. In addition, anti-HA mAb also detected the Pb115 C-terminal HA tag on gametes and ookinetes in a similar way. Since several gamete membrane proteins such as P48/45 and P230 are primary TBV candidates, the membrane expression of Pb115 during sexual stages prompted us to investigate its transmission-blocking potential. We generated the recombinant MFS domain from yeast and immunized mice to study its TRA using both *in vitro* ookinete conversion and *in vivo* mosquito feeding assays. Antibodies against the synthetic protein could recognize Pb115 in Western blot and IFA, suggesting that the recombinant protein possessed, at least in part, properly folded epitopes. Antisera against rPb115 significantly reduced zygote formation and ookinete conversion. Direct feeding of mosquitoes on rPb115-immunized mice showed 44% reduction in prevalence and 39% reduction in oocyst intensity as compared to those fed on non-immunized mice. Compared with the TRA of other gamete membrane proteins such as P48/45 (Outchkourov et al., 2008), P230 (Williamson et al., 1995), and HAP2 (Blagborough and Sinden, 2009), the extents of reduction are rather modest and further refinement of the recombinant protein covering important epitopes may help improve the TB activity of Pb115 (Deore et al., 2019). The expression of Pb115 on both gametes and ookinetes suggests that it might be a target for both pre- and post-fertilization immunity. In addition, studies on P115 in human malaria parasites are warranted, given the high degree of conservation of P115 in different *Plasmodium* species.

## Data Availability Statement

The raw data supporting the conclusions of this manuscript will be made available by the authors, without undue reservation, to any qualified researcher.

## Ethics Statement

This study was carried out in accordance with the recommendations of the guidelines established by China Medical University, Animal Welfare and Research Ethics Committee. The protocol was approved by the Animal Care and Use Committee of China Medical University.

## Author Contributions

FL performed the main experiments and wrote the manuscript. QL, CY, YZ, YW, HM, YQ, YJ, and JM provided laboratory assistance. JM took part in most supplemental experiments and revised the manuscript. YC designed the experiments. LC supervised the study and revised the manuscript.

## Conflict of Interest

The authors declare that the research was conducted in the absence of any commercial or financial relationships that could be construed as a potential conflict of interest.

## Footnotes

1 http://www.plasmodb.org

## References

[B1] Bhatt S. Weiss D. J. Cameron E. Bisanzio D. Mappin B. Dalrymple U. (2015). The effect of malaria control on *Plasmodium falciparum* in Africa between 2000 and 2015. *Nature* 526 207–211. 10.1038/nature15535 26375008 PMC4820050

[B2] Blagborough A. M. Sinden R. E. (2009). *Plasmodium berghei* HAP2 induces strong malaria transmission-blocking immunity in vivo and in vitro. *Vaccine* 27 5187–5194. 10.1016/j.vaccine.2009.06.069 19596419

[B3] Braks J. A. Franke-Fayard B. Kroeze H. Janse C. J. Waters A. P. (2006). Development and application of a positive-negative selectable marker system for use in reverse genetics in *Plasmodium*. *Nucleic Acids Res.* 34:e39. 10.1093/nar/gnj033 16537837 PMC1401515

[B4] Chan J. A. Fowkes F. J. Beeson J. G. (2014). Surface antigens of *Plasmodium falciparum*-infected erythrocytes as immune targets and malaria vaccine candidates. *Cell. Mol. Life Sci.* 71 3633–3657. 10.1007/s00018-014-1614-3 24691798 PMC4160571

[B5] Chowdhury D. R. Angov E. Kariuki T. Kumar N. (2009). A potent malaria transmission blocking vaccine based on codon harmonized full length Pfs48/45 expressed in *Escherichia coli*. *PLoS One* 4:e6352. 10.1371/journal.pone.0006352 19623257 PMC2709910

[B6] Delves M. J. Angrisano F. Blagborough A. M. (2018). Antimalarial transmission-blocking interventions: past, present, and future. *Trends Parasitol.* 34 735–746. 10.1016/j.pt.2018.07.001 30082147

[B7] Dempsey E. Prudencio M. Fennell B. J. Gomes-Santos C. S. Barlow J. W. Bell A. (2013). Antimitotic herbicides bind to an unidentified site on malarial parasite tubulin and block development of liver-stage *Plasmodium parasites*. *Mol. Biochem. Parasitol.* 188 116–127. 10.1016/j.molbiopara.2013.03.001 23523992

[B8] Deore S. Kumar A. Kumar S. Mittal E. Lotke A. Musti K. (2019). Erythrocyte binding ligand region VI specific IgA confers tissue protection in malaria infection. *Mol. Biol. Rep.* 46 3801–3808. 10.1007/s11033-019-04822-7 31012028

[B9] Ecker A. Bushell E. S. Tewari R. Sinden R. E. (2008). Reverse genetics screen identifies six proteins important for malaria development in the mosquito. *Mol. Microbiol.* 70 209–220. 10.1111/j.1365-2958.2008.06407.x 18761621 PMC2658712

[B10] Eksi S. Czesny B. van Gemert G. J. Sauerwein R. W. Eling W. Williamson K. C. (2006). Malaria transmission-blocking antigen, Pfs230, mediates human red blood cell binding to exflagellating male parasites and oocyst production. *Mol. Microbiol.* 61 991–998. 10.1111/j.1365-2958.2006.05284.x 16879650

[B11] Gardner M. J. Hall N. Fung E. White O. Berriman M. Hyman R. W. (2002). Genome sequence of the human malaria parasite *Plasmodium falciparum*. *Nature* 419 498–511. 12368864 10.1038/nature01097 PMC3836256

[B12] Guttery D. S. Poulin B. Ramaprasad A. Wall R. J. Ferguson D. J. Brady D. (2014). Genome-wide functional analysis of plasmodium protein phosphatases reveals key regulators of parasite development and differentiation. *Cell Host Microbe.* 16 128–140. 10.1016/j.chom.2014.05.020 25011111 PMC4094981

[B13] Hall N. Karras M. Raine J. D. Carlton J. M. Kooij T. W. Berriman M. (2005). A comprehensive survey of the *Plasmodium* life cycle by genomic, transcriptomic, and proteomic analyses. *Science* 307 82–86. 10.1126/science.1103717 15637271

[B14] Hirai M. Arai M. Mori T. Miyagishima S. Y. Kawai S. Kita K. (2008). Male fertility of malaria parasites is determined by GCS1, a plant-type reproduction factor. *Curr. Biol.* 18 607–613. 10.1016/j.cub.2008.03.045 18403203

[B15] Kaslow D. C. Quakyi I. A. Syin C. Raum M. G. Keister D. B. Coligan J. E. (1988). A vaccine candidate from the sexual stage of human malaria that contains EGF-like domains. *Nature* 333 74–76. 10.1038/333074a0 3283563

[B16] Kocken C. H. Jansen J. Kaan A. M. Beckers P. J. Ponnudurai T. Kaslow D. C. (1993). Cloning and expression of the gene coding for the transmission blocking target antigen Pfs48/45 of *Plasmodium falciparum*. *Mol. Biochem. Parasitol.* 61 59–68. 10.1016/0166-6851(93)90158-t 8259133

[B17] Kou X. Zheng W. Du F. Liu F. Wang M. Fan Q. (2016). Characterization of a *Plasmodium berghei* sexual stage antigen PbPH as a new candidate for malaria transmission-blocking vaccine. *Parasit. Vectors* 9:190. 10.1186/s13071-016-1459-8 27038925 PMC4818878

[B18] Lasonder E. Rijpma S. R. van Schaijk B. C. Hoeijmakers W. A. Kensche P. R. Gresnigt M. S. (2016). Integrated transcriptomic and proteomic analyses of *P. falciparum* gametocytes: molecular insight into sex-specific processes and translational repression. *Nucleic Acids Res.* 44 6087–6101. 10.1093/nar/gkw536 27298255 PMC5291273

[B19] Law C. J. Maloney P. C. Wang D. N. (2008). Ins and outs of major facilitator superfamily antiporters. *Annu. Rev. Microbiol.* 62 289–305. 10.1146/annurev.micro.61.080706.093329 18537473 PMC2612782

[B20] Lee S. M. Wu C. K. Plieskatt J. L. Miura K. Hickey J. M. King C. R. (2017). An N-terminal Pfs230 domain produced in baculovirus as a biological active transmission-blocking vaccine candidate. *Clin. Vaccine Immunol.* 24:e00140-17. 10.1128/CVI.00140-17 28747311 PMC5629673

[B21] Liu F. Li L. Zheng W. He Y. Wang Y. Zhu X. (2018). Characterization of *Plasmodium berghei* Pbg37 as both a pre- and postfertilization antigen with transmission-blocking potential. *Infect. Immun.* 86:e00785-17. 10.1128/IAI.00785-17 29866905 PMC6056874

[B22] Liu Y. Tewari R. Ning J. Blagborough A. M. Garbom S. Pei J. (2008). The conserved plant sterility gene HAP2 functions after attachment of fusogenic membranes in *Chlamydomonas* and *Plasmodium gametes*. *Genes Dev.* 22 1051–1068. 10.1101/gad.1656508 18367645 PMC2335326

[B23] Molina-Cruz A. Garver L. S. Alabaster A. Bangiolo L. Haile A. Winikor J. (2013). The human malaria parasite Pfs47 gene mediates evasion of the mosquito immune system. *Science* 340 984–987. 10.1126/science.1235264 23661646 PMC3807741

[B24] Nikolaeva D. Draper S. J. Biswas S. (2015). Toward the development of effective transmission-blocking vaccines for malaria. *Expert Rev. Vaccines* 14 653–680. 10.1586/14760584.2015.993383 25597923

[B25] Outchkourov N. S. Roeffen W. Kaan A. Jansen J. Luty A. Schuiffel D. (2008). Correctly folded Pfs48/45 protein of *Plasmodium falciparum* elicits malaria transmission-blocking immunity in mice. *Proc. Natl. Acad. Sci. U.S.A.* 105 4301–4305. 10.1073/pnas.0800459105 18332422 PMC2393789

[B26] Sagara I. Healy S. A. Assadou M. H. Gabriel E. E. Kone M. Sissoko K. (2018). Safety and immunogenicity of Pfs25H-EPA/Alhydrogel, a transmission-blocking vaccine against *Plasmodium falciparum*: a randomised, double-blind, comparator-controlled, dose-escalation study in healthy malian adults. *Lancet Infect. Dis.* 18 969–982. 10.1016/S1473-3099(18)30344-X 30061051 PMC6287938

[B27] Sauerwein R. W. Bousema T. (2015). Transmission blocking malaria vaccines: assays and candidates in clinical development. *Vaccine* 33 7476–7482. 10.1016/j.vaccine.2015.08.073 26409813

[B28] Shimp R. L. Jr. Rowe C. Reiter K. Chen B. Nguyen V. Aebig J. (2013). Development of a Pfs25-EPA malaria transmission blocking vaccine as a chemically conjugated nanoparticle. *Vaccine* 31 2954–2962. 10.1016/j.vaccine.2013.04.034 23623858 PMC3683851

[B29] Tachibana M. Miura K. Takashima E. Morita M. Nagaoka H. Zhou L. (2019). Identification of domains within Pfs230 that elicit transmission blocking antibody responses. *Vaccine* 37 1799–1806. 10.1016/j.vaccine.2019.02.021 30824357 PMC6708081

[B30] Talaat K. R. Ellis R. D. Hurd J. Hentrich A. Gabriel E. Hynes N. A. (2016). Safety and immunogenicity of Pfs25-EPA/Alhydrogel(R), a transmission blocking vaccine against *Plasmodium falciparum*: an open label study in malaria naive adults. *PLoS One* 11:e0163144. 10.1371/journal.pone.0163144 27749907 PMC5066979

[B31] Tewari R. Straschil U. Bateman A. Bohme U. Cherevach I. Gong P. (2010). The systematic functional analysis of *Plasmodium protein* kinases identifies essential regulators of mosquito transmission. *Cell Host Microbe.* 8 377–387. 10.1016/j.chom.2010.09.006 20951971 PMC2977076

[B32] Tonkin C. J. van Dooren G. G. Spurck T. P. Struck N. S. Good R. T. Handman E. (2004). Localization of organellar proteins in *Plasmodium falciparum* using a novel set of transfection vectors and a new immunofluorescence fixation method. *Mol. Biochem. Parasitol.* 137 13–21. 10.1016/j.molbiopara.2004.05.009 15279947

[B33] Tsuboi T. Kaslow D. C. Gozar M. M. Tachibana M. Cao Y. M. Torii M. (1998). Sequence polymorphism in two novel *Plasmodium* vivax ookinete surface proteins, Pvs25 and Pvs28, that are malaria transmission-blocking vaccine candidates. *Mol. Med.* 4 772–782. 10.1007/bf03401770 9990863 PMC2230397

[B34] van Dijk M. R. Janse C. J. Thompson J. Waters A. P. Braks J. A. Dodemont H. J. (2001). A central role for P48/45 in malaria parasite male gamete fertility. *Cell* 104 153–164. 10.1016/s0092-8674(01)00199-4 11163248

[B35] van Dijk M. R. van Schaijk B. C. Khan S. M. van Dooren M. W. Ramesar J. Kaczanowski S. (2010). Three members of the 6-cys protein family of *Plasmodium* play a role in gamete fertility. *PLoS Pathog.* 6:e1000853. 10.1371/journal.ppat.1000853 20386715 PMC2851734

[B36] van Schaijk B. C. van Dijk M. R. van de Vegte-Bolmer M. van Gemert G. J. van Dooren M. W. Eksi S. (2006). Pfs47, paralog of the male fertility factor Pfs48/45, is a female specific surface protein in *Plasmodium falciparum*. *Mol. Biochem. Parasitol.* 149 216–222. 10.1016/j.molbiopara.2006.05.015 16824624

[B37] Vaughan J. A. (2007). Population dynamics of *Plasmodium sporogony*. *Trends Parasitol.* 23 63–70. 10.1016/j.pt.2006.12.009 17188574

[B38] World Health Organization [WHO] (2018). *World Malaria Report 2017.* Geneva: World Health Organization.

[B39] Williamson K. C. Criscio M. D. Kaslow D. C. (1993). Cloning and expression of the gene for *Plasmodium falciparum* transmission-blocking target antigen, Pfs230. *Mol. Biochem. Parasitol.* 58 355–358. 10.1016/0166-6851(93)90058-6 8479460

[B40] Williamson K. C. Keister D. B. Muratova O. Kaslow D. C. (1995). Recombinant Pfs230, a *Plasmodium falciparum* gametocyte protein, induces antisera that reduce the infectivity of *Plasmodium falciparum* to mosquitoes. *Mol. Biochem. Parasitol.* 75 33–42. 10.1016/0166-6851(95)02507-3 8720173

[B41] Wu Y. Sinden R. E. Churcher T. S. Tsuboi T. Yusibov V. (2015). Development of malaria transmission-blocking vaccines: from concept to product. *Adv. Parasitol.* 89 109–152. 10.1016/bs.apar.2015.04.001 26003037

[B42] Yan N. (2015). Structural biology of the major facilitator superfamily transporters. *Annu. Rev. Biophys.* 44 257–283. 10.1146/annurev-biophys-060414-033901 26098515

[B43] Zheng W. Kou X. Du Y. Liu F. Yu C. Tsuboi T. (2016). Identification of three ookinete-specific genes and evaluation of their transmission-blocking potentials in *Plasmodium berghei*. *Vaccine* 34 2570–2578. 10.1016/j.vaccine.2016.04.011 27083421 PMC4864593

[B44] Zheng W. Liu F. He Y. Liu Q. Humphreys G. B. Tsuboi T. (2017). Functional characterization of *Plasmodium berghei* PSOP25 during ookinete development and as a malaria transmission-blocking vaccine candidate. *Parasit. Vectors* 10:8. 10.1186/s13071-016-1932-4 28057055 PMC5217559

[B45] Zhu X. Sun L. He Y. Wei H. Hong M. Liu F. (2019). Plasmodium berghei serine/threonine protein phosphatase PP5 plays a critical role in male gamete fertility. *Int. J. Parasitol.* 49 685–695. 10.1016/j.ijpara.2019.03.007 31202684 PMC8889123

