# Supplementary material for: An MFS-Domain Protein Pb115 Plays a Critical Role in Gamete Fertilization of the Malaria Parasite Plasmodium berghei
Source: Front Microbiol. 2019 Sep 20;10:2193. doi: 10.3389/fmicb.2019.02193 (PMC6764285; doi:10.3389/fmicb.2019.02193)
Supplement: Supplementary file 1 [file Data_Sheet_1.PDF]

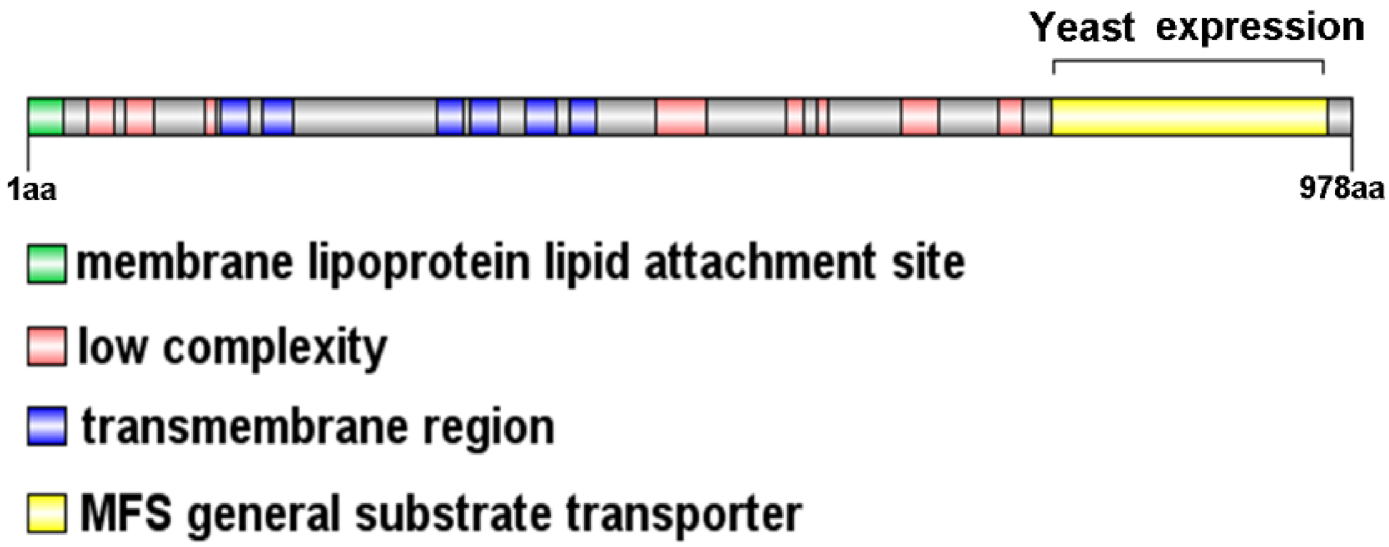

FIGURE S1 Bioinformatic analyses of Pb115. Pb115 contains a membrane lipoprotein lipid attachment site profile at its N-terminus and a MSF general substrate transporter domain near the C terminus. Yeast expression shows the fragment (amino acids 756 – 960) that was expressed in yeast.

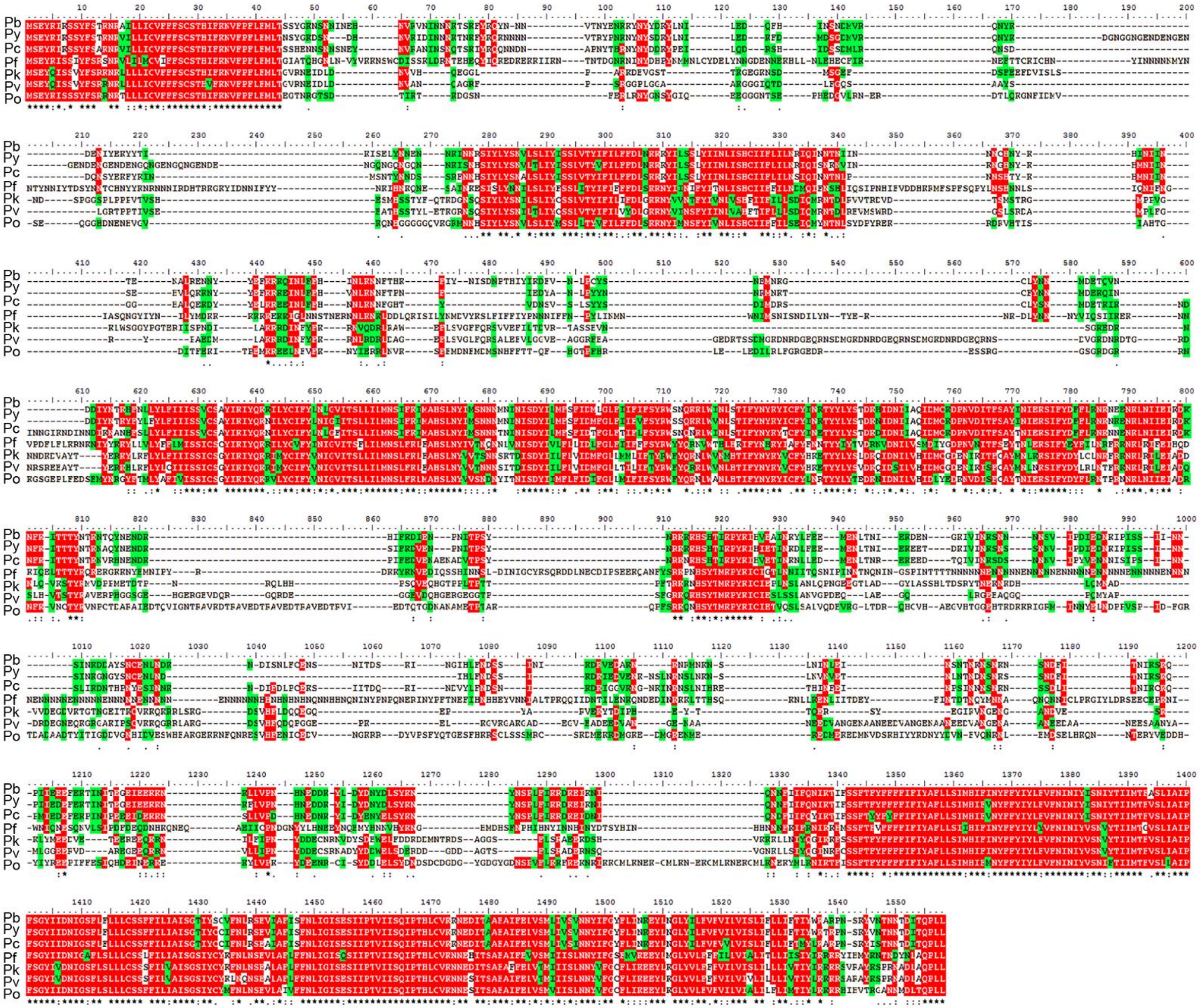

FIGURE S2 Alignment of protein sequences of P115 in seven *Plasmodium* species: *P. berghei* (*Pb*), *P. yoelii* (*Py*), *P. chabaudi* (*Pc*), *P. falciparum, P. knowlesi* (*Pk*), *P. vivax* (*Pv*) and *P. ovale* (*Po*). Amino acids are marked in red for identity and green for similarity across seven species.

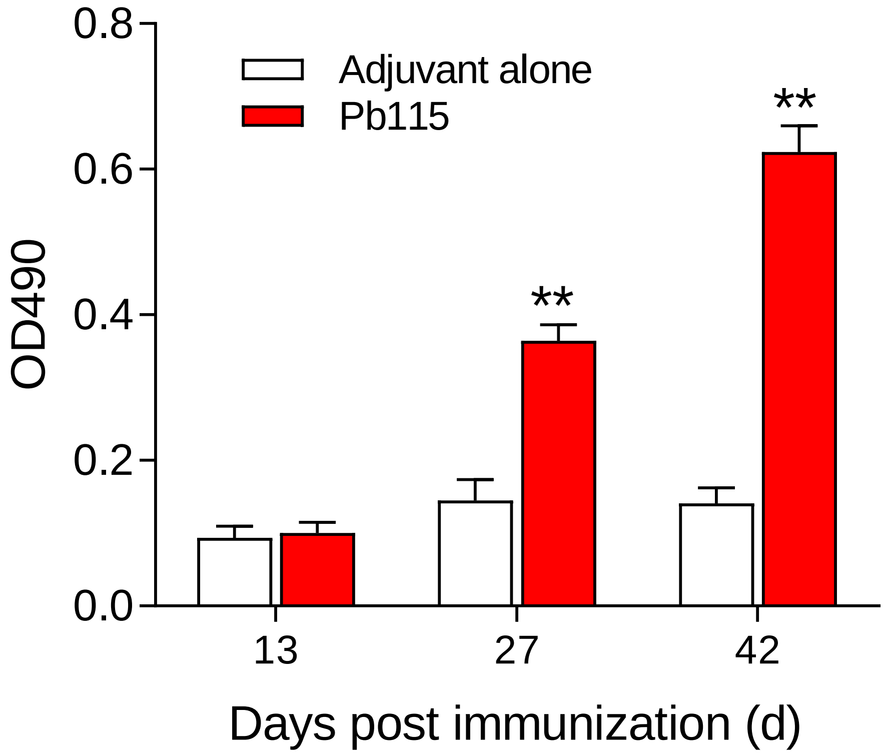

FIGURE S3 Enzyme-linked immunoassay (ELISA) showing Pb115 antibody titers after immunization. Serum samples were collected before the day of each immunization and after two weeks of the third immunization. ** indicates significant difference between Pb115-immunized mice and adjuvant control mice ($P < 0.01$, Student's *t* test)

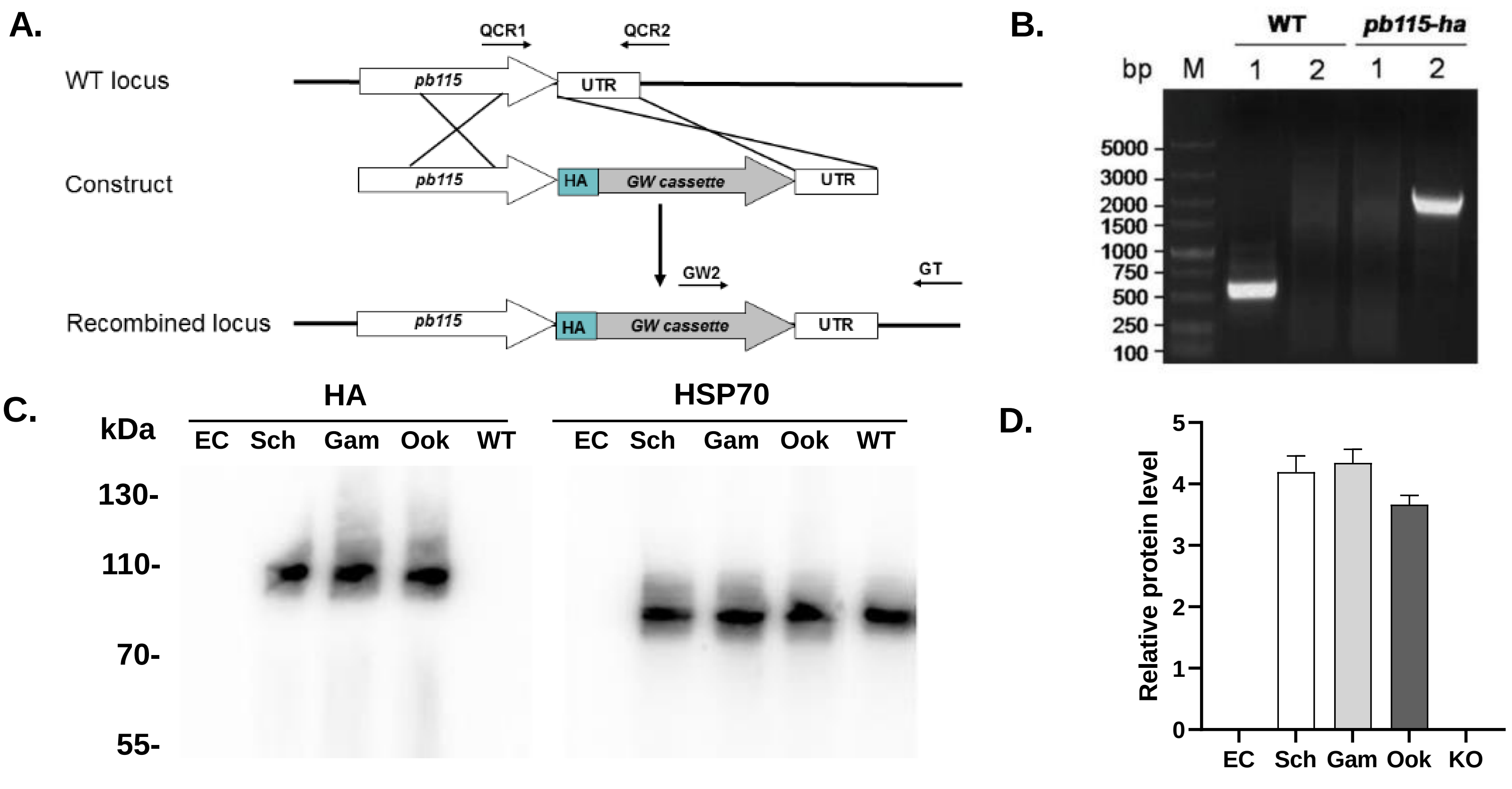

FIGURE S4 Tagging of Pb115 with an HA-tag and detection of Pb115 expression. **(A)** HA-tagged parasite lines (*pb115-ha*) were generated using double-crossover homologous recombination. The scheme shows the wild-type (WT) pb115 locus, the plasmid construct and recombined locus with the locations of the primers (QCR1, QCR2, GW2, and GT) used for integration-specific PCR marked. **(B)** Integration-specific PCR was used to confirm the generation of HA-tagged lines. Lane 1: primers QCR1 + QCR2 (657 bp); Lane 2: primer GW2 + GT (2177 bp). GW cassette contains the *yfcu* cassette and *dhfr* cassette. **(C)** Western blot of Pb115 in asexual- and sexual-stage parasites. Lysates from purified Pb115-HA schizonts (Sch), gametocytes (Gam), ookinetes (Ook), and wild-type ookinetes (WT) at 10 μg/lane proteins were separated by SDS-PAGE and probed with anti-HA monoclonal antibody (1:1000). Protein loading was estimated using an anti-Hsp70 serum (1:1000). Non-infected erythrocytes (EC) were used as negative control. **(D)** Analysis of protein band intensities in **(C)** using ImageJ to show the relative Pb115 protein levels in different stages. Relative expression level indicates Pb115 protein band intensity normalized against the HSP70 control.

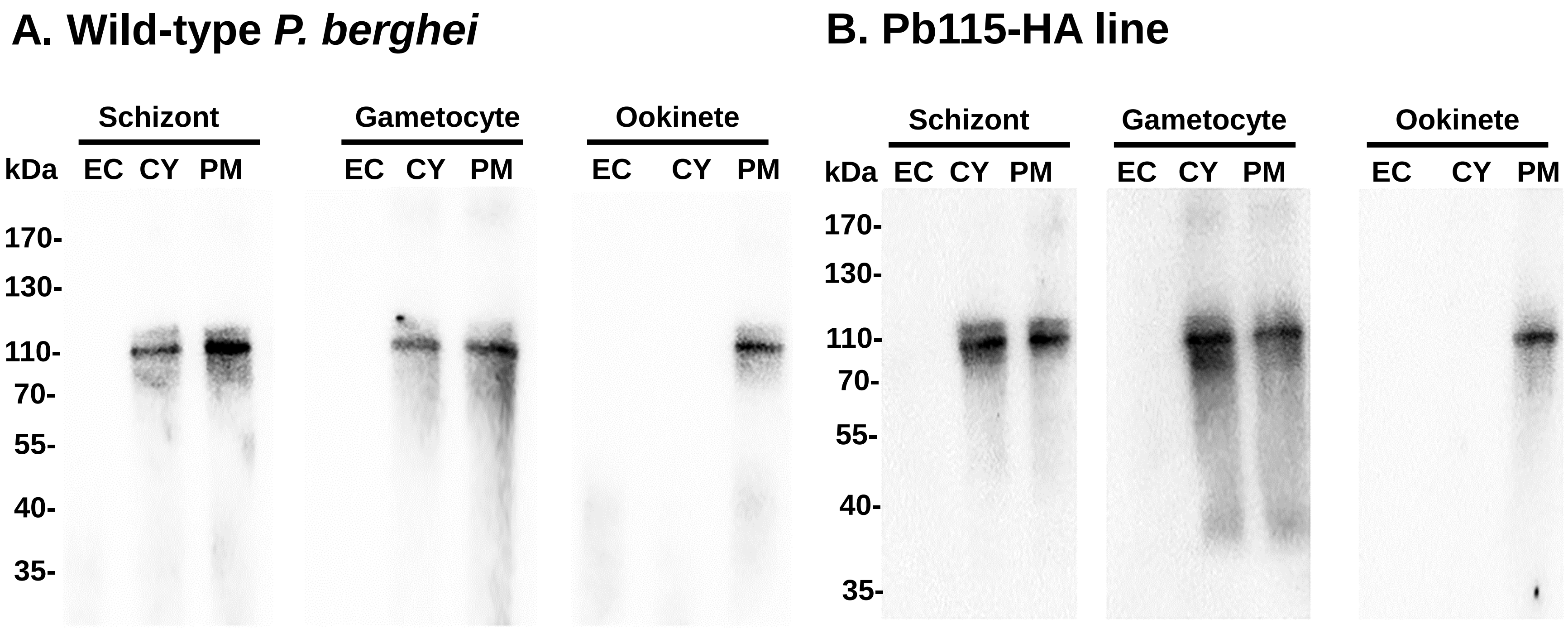

FIGURE S5 Western blots of Pb-115 in protein extracts from wild-type **(A)** and Pb115-HA **(B)** lines. Protein extract of non-infected erythrocytes (EC), cytoplasm fraction (CY), and plasma membrane fraction (PM) of schizonts, gametocytes and ookinetes were separated by SDS-PAGE. The blots were probed with anti-Pb115 sera **(A)** or anti-HA monoclonal antibody **(B)**.

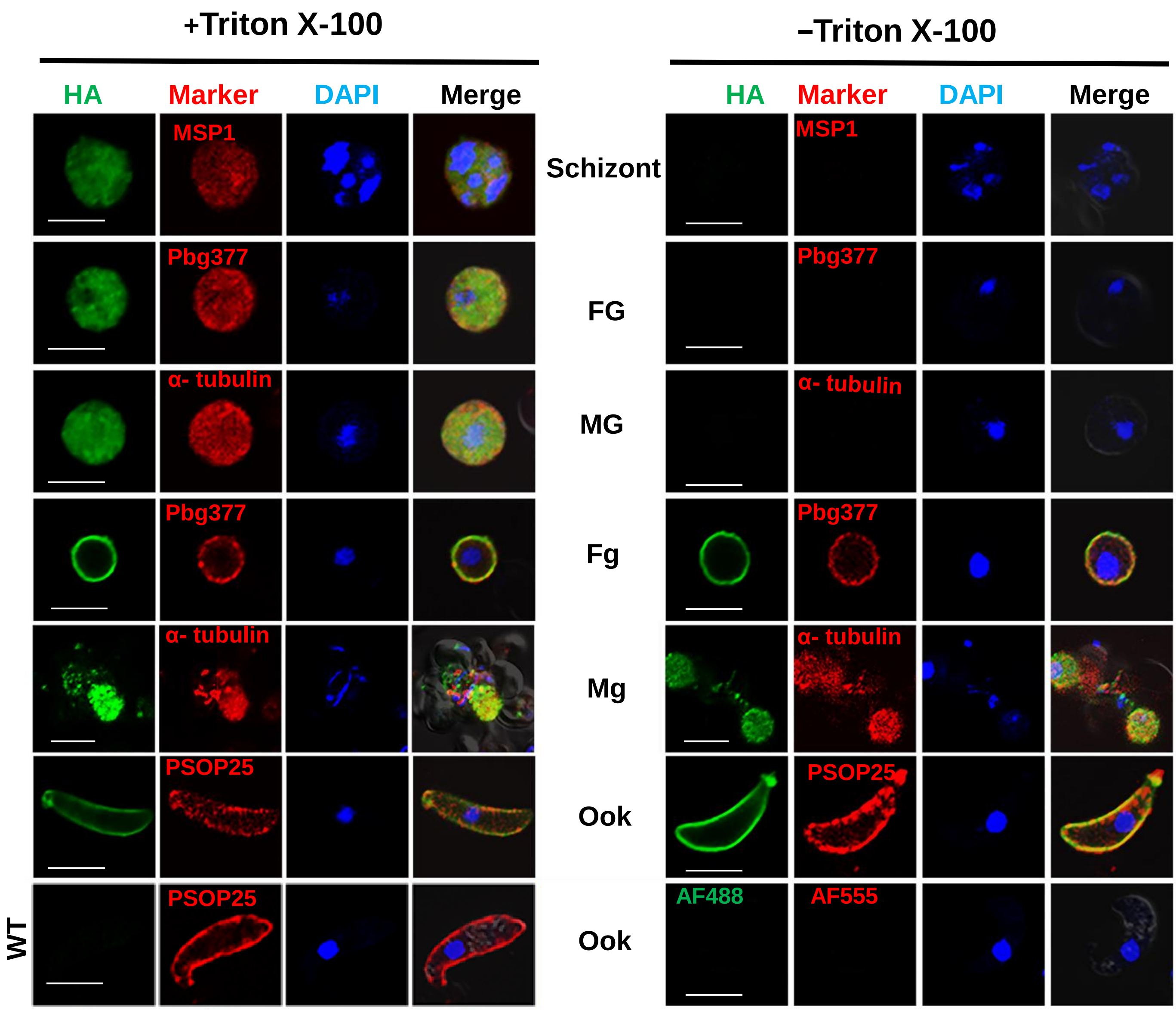

FIGURE S6 IFA showing localization of Pb115 in different developmental stages in the Pb115-HA parasite line. **(A)** Cells were permeabilized with or without 0.1% Triton X-100. Primary antibodies are anti-HA monoclonal antibody (green) and rabbit antisera specific for PbMSP1, Pbg377, α-tubulin and PSOP25 (red) to show individual developmental stages. Secondary antibodies are Alexa Fluor 488-conjugated goat-anti-mouse IgG (green) and Alexa Flour 555-conjugated goat-anti-rabbit IgG (red). Nuclei were counter-stained with DAPI (blue). FG – Female gametocyte, MG – Male gametocyte, Fg – Female gamete, MG – Male gamete, Ook – ookinete. Bar, 5 μm. The bottom panel labeled with WT shows the lack of reactivity of the WT ookinete with the anti-HA antibodies.

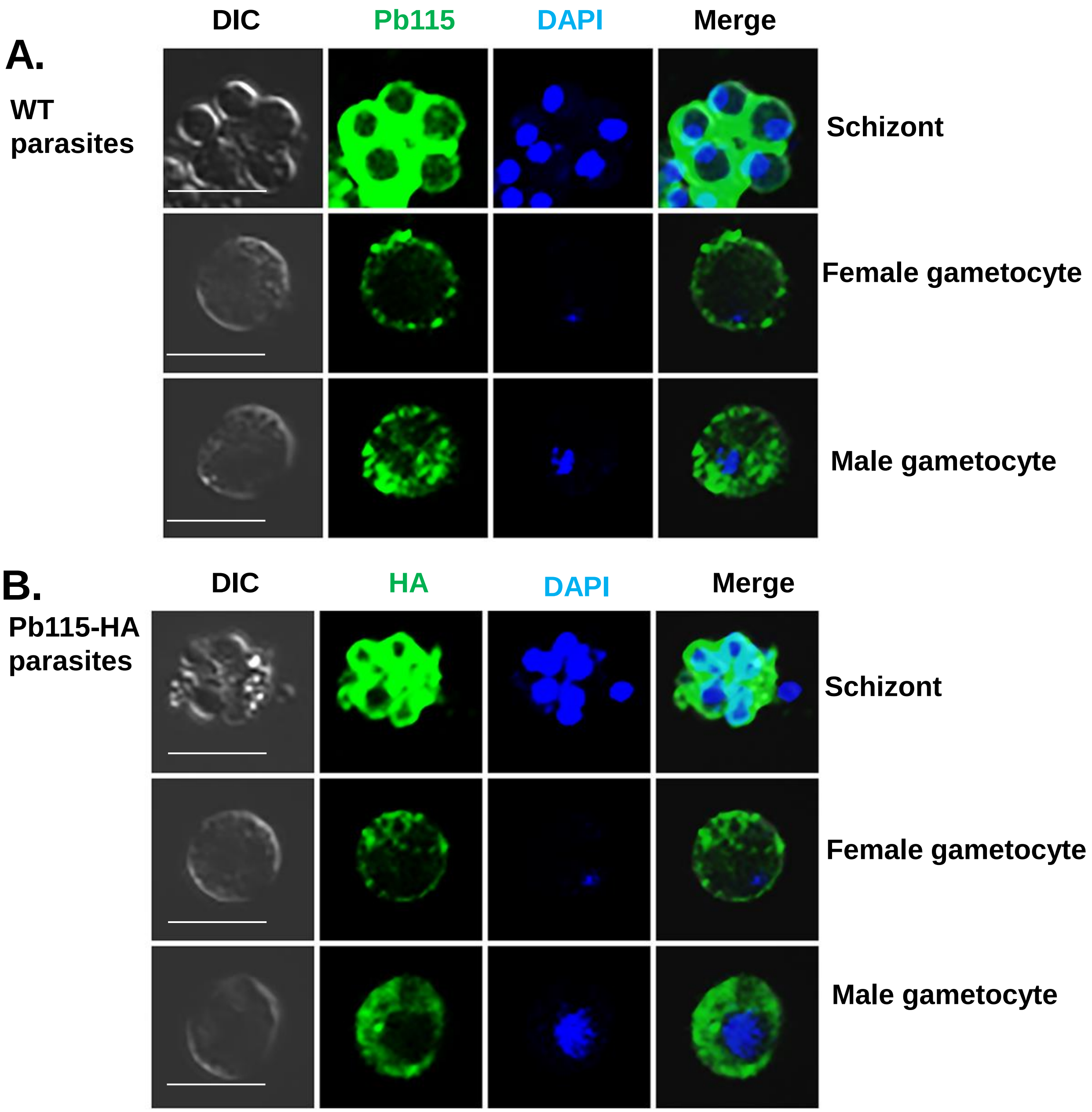

FIGURE S7 Localization of Pb115 in WT **(A)** and Pb115-HA parasites **(B)**. Parasites were stripped from the enveloping erythrocyte and parasitophorous vacuole membranes by 0.05% saponin treatment prior to fixation without further permeabilization. Mouse anti-Pb115 sera (1:500) **(A)** and monoclonal anti-HA antibody (1:1000) **(B)** were the primary antibodies, while Alexa Fluor 488-conjugated goat-anti-mouse IgG (green) was the secondary antibodies. The nuclei were stained by DAPI (1 μg/mL) (blue). Merge, DIC + Alexa Flour 488 + DAPI. Bar, 5 μm.

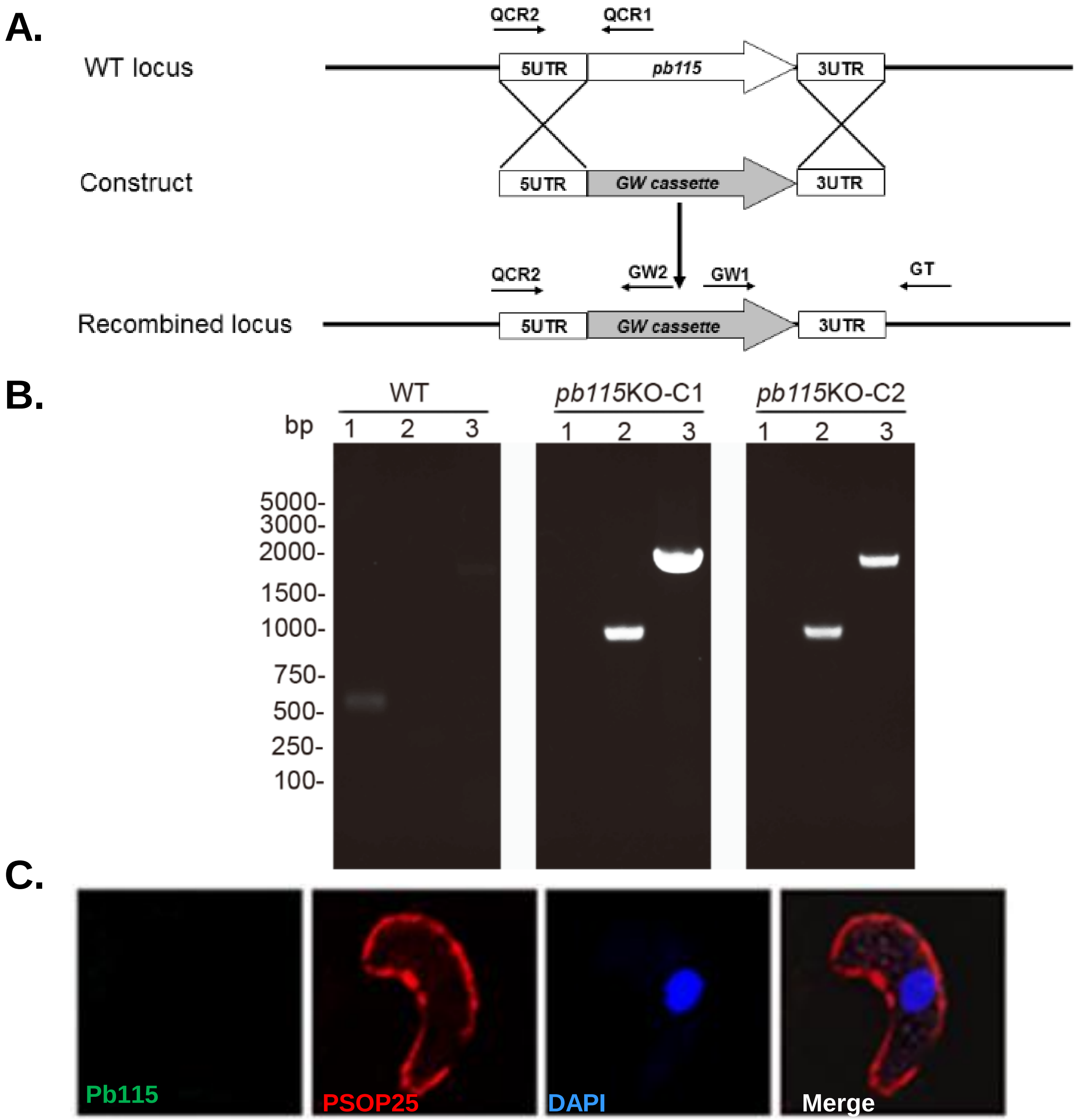

FIGURE S8 Generation of Pb115 deletion parasites. **(A)** Scheme showing knockout of the *Pb115* gene by homologous recombination. Primers and locations are indicated as arrows. **(B)** Integration-specific PCR was used to confirm the generation of *pb115KO* knockout lines. *pb115KO-C1 and pb115KO-C2* are two KO lines generated in two independent transfection experiments. Lane 1: primers QCR1 + QCR2 (565 bp); Lane 2: primer QCR2 + GW2 (1012 bp); Lane 3: primer GW1 + GT (2035 bp). GW cassette contains the *yfcu* cassette and *dhfr* cassette. **(C)** IFA showing the lack of detection of Pb115 on the surface of ookinete in *pb115KO-C1*. Primary antibodies include mouse anti-Pb115 serum and rabbit antisera for PSOP25. Secondary antibodies are Alexa Fluor 488-conjugated goat-anti-mouse IgG (green) and Alexa Flour 555-conjugated goat-anti-rabbit IgG (red). Nucleus was stained with DAPI.

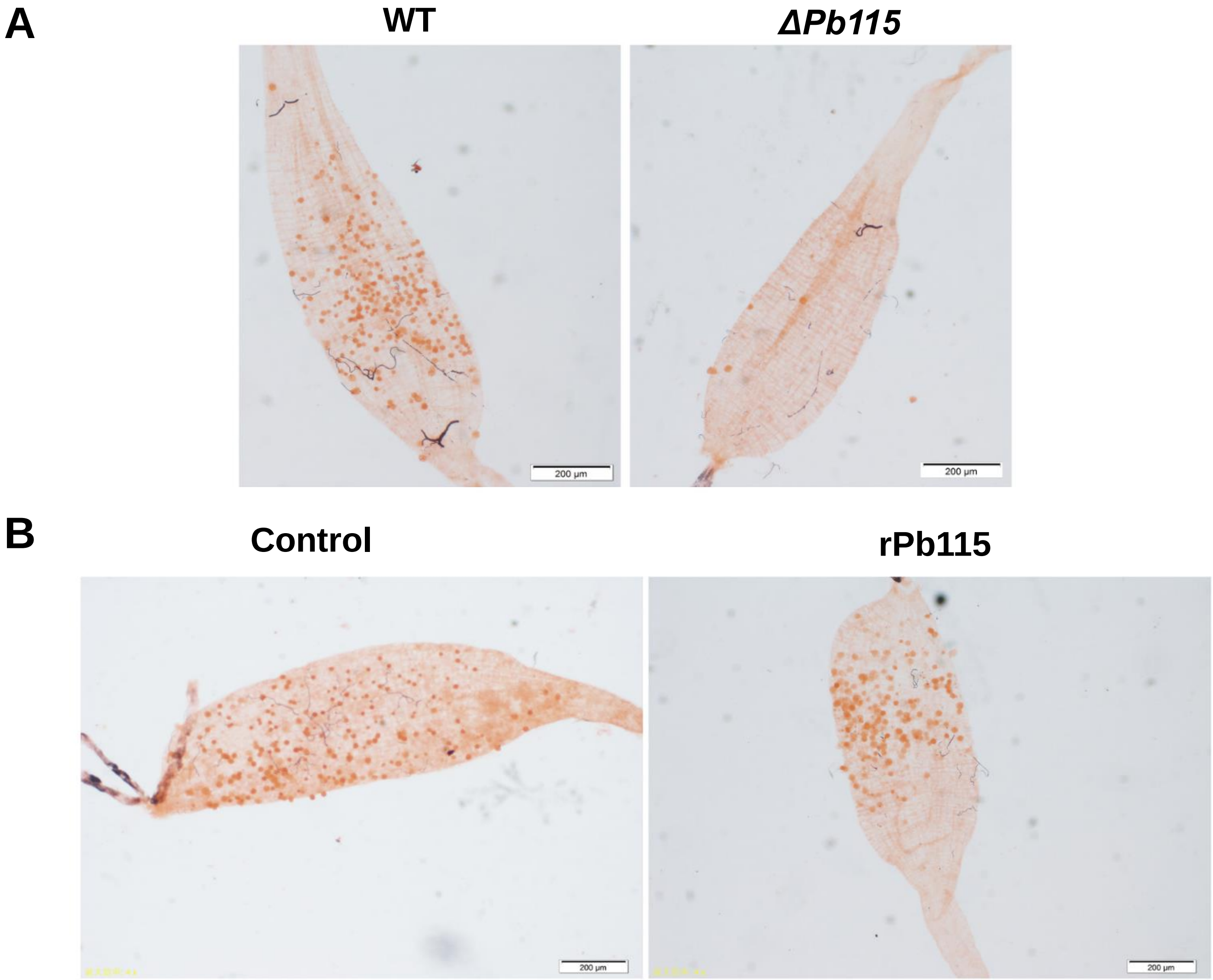

FIGURE S9 Representative images of oocyst densities on mosquito midguts. **(A)** The oocyst densities for mosquitoes fed on WT- and *ΔPb115*-infected mice. **(B)** The oocyst densities for mosquitoes fed on control and rPb115-immunized mice. All images were obtained under a 4 × objective.
