# Supplementary material for: An MFS-Domain Protein Pb115 Plays a Critical Role in Gamete Fertilization of the Malaria Parasite Plasmodium berghei
Source: Front Microbiol. 2019 Sep 20;10:2193. doi: 10.3389/fmicb.2019.02193 (PMC6764285; doi:10.3389/fmicb.2019.02193)
Supplement: Supplementary file 2 [file Table_1.DOC]

| **Table S1:Integration-specific primers for transgenic parasites** | | |
| --- | --- | --- |
| Purpose | Primer name | Sequence |
| HA-Tag | QCR1 | ACCATATATTGGAAGGCAAGACCA |
|  | QCR2 | ACACCCCCGGGTTTCCTGACA |
|  | GT | AAGATGACTTTCGTCGCAGA |
| Δ*pb115* line integration-specific PCR | QCR1 | TGTGTGTTGAGCATGAAAAA |
|  | QCR2 | AGTGCCAAACTAAGTTCGAACA |
|  | GT | AAGATGACTTTCGTCGCAGA |
| GW Cassette | GW1 | CATACTAGCCATTTTATGTG |
|  | GW2 | CTTTGGTGACAGATACTAC |
| Δ*pb47* line 5’ and 3’ recombinant fragment | 5U-F | CGGGGCCCAACAACATTCGCATCCTCTCTG |
|  | 5U-R | TCCCCGCGGTAGATACAGAAAACGGAACGAC |
|  | 3U-F | CCCTCGAGAGTAACATTTCGTATGGGTA |
|  | 3U-R | CAGCGGCCGCTGATGGATGAAGAATGACCG |
| Δ*pb47* line integration-specific PCR | P1 | TATACCCTTTACCAATTCCC |
|  | P2 | ATAGAAGCCCCTGTGAAGCC |
|  | P5 | TGGGTCATCCTGTTTAGGTAT |
| Δ*pb48* line 5’ and 3’ recombinant fragment | 5U-F | CGGGGCCC CTATTAAGTAACAGTATCCCCTA |
|  | 5U-R | TCCCCGCGGGTTACAAGCCATAATAGAAT |
|  | 3U-F | CCGCTCGAGGTGGGTATTGACGTATGCTT |
|  | 3U-R | CAGCGGCCGCATCCACATAACGCTAATGAT |
| Δ*pb48* line integration-specific PCR | P1 | GGGACATTCTAAACAATCGCAG |
|  | P2 | ATGCGACTTTTCCACCGAAG |
|  | P5 | GGCTTCACAGGGGCTTCTAT |
| PL0034 Vector | P3 | CTGGTGCTTTGAGGGGTGAG |
|  | P4 | TTGTTACTGGTGCCCTCGAC |
